# Ventricular wall stress and wall shear stress homeostasis predicts cardiac remodeling during pregnancy: A modeling study

**DOI:** 10.1002/cnm.3536

**Published:** 2021-10-18

**Authors:** Giulia Comunale, Francesca M. Susin, Jonathan P. Mynard

**Affiliations:** ^1^ Cardiovascular Fluid Dynamics Laboratory HER, Department of Civil, Environmental and Architectural Engineering University of Padova Padova Italy; ^2^ Heart Research Murdoch Children's Research Institute Parkville Victoria Australia; ^3^ Department of Pediatrics University of Melbourne Parkville Victoria Australia; ^4^ Department of Biomedical Engineering University of Melbourne Parkville Victoria Australia

**Keywords:** cardiac remodeling, cardiovascular modeling, lumped parameter models, pregnancy

## Abstract

Pregnancy is a unique and dynamic process characterized by significant changes in the maternal cardiovascular system that are required to satisfy the increased maternal and fetal metabolic demands. Profound structural and hemodynamic adaptations occur during healthy pregnancy that allows the mother to maintain healthy hemodynamics and provide an adequate uteroplacental blood circulation to ensure physiological fetal development. Investigating these adaptations is crucial for understanding the physiology of pregnancy and may provide important insights for the management of high‐risk pregnancies. However, no previous modeling studies have investigated the maternal cardiac structural changes that occur during gestation. This study, therefore, had two aims. The first was to develop a lumped parameter model of the whole maternal circulation that is suitable for studying global hemodynamics and cardiac function at different stages of gestation. The second was to test the hypothesis that myofiber stress and wall shear stress homeostasis principles can be used to predict cardiac remodeling that occurs during normal pregnancy. Hemodynamics and cardiac variables predicted from simulations with and without controlled cardiac remodeling algorithms were compared and evaluated with reference clinical data. While both models reproduced the hemodynamic variations that arise in pregnancy, importantly, we show that the structural changes that occur with pregnancy could be predicted by assuming invariant homeostatic “target” values of myocardial wall stress and chamber wall shear stress.

## Graphical abstract

- First model to simulate both cardiac remodeling and changes in hemodynamics during a normal pregnancy.
- We developed a lumped model of the maternal circulation suitable for studying global hemodynamics and cardiac function at different stages of gestation.
- Imposing simple myofiber stress and wall shear stress homeostasis principles predicted cardiac remodeling during pregnancy.
- The model reproduces gestational cardiovascular adaptation during pregnancy, considering the volume overload condition, and physiological remodeling.

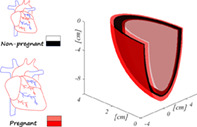

**Abbreviations**
CO: cardiac output
CRA: hemodynamic model coupled with the controlled remodeling algorithm
*h*
: thickness
LA: left atrium
LAD: left atrial diameter
LV: left ventricle
LVEDD: end‐diastolic left ventricular diameter
LVM: left ventricular mass
NCRA: hemodynamic model coupled with no controlled remodeling algorithm
NPC: non‐pregnant case
*r*
: radius
RA: right atrium
RAAS: renin‐angiotensin‐aldosterone system
RV: right ventricle
RWT: relative wall thickness
$\sigma_{f}$
: myofiber stress
SV: stroke volume
SVR: systemic vascular resistance
$\sigma_{\mathrm{wss}}$
: wall shear stress
T1: first trimester
T2: second trimester
T3: third trimester
$V_{\mathrm{myo}}$
: myocardial volume
$V_{{\mathrm{TOT}}_{u}}$
: unstressed volume
$V_{{\mathrm{TOT}}_{S}}$
: stressed volume

## 1 INTRODUCTION

Pregnancy is a unique and dynamic process characterized by significant changes in the maternal cardiovascular system that are required to satisfy maternal and fetal metabolic demands. Significant structural and hemodynamic adaptations occur during healthy pregnancy that allows the mother to maintain healthy hemodynamics and guarantee an adequate uteroplacental blood supply to support fetal development. Starting from early pregnancy, increased heart rate and stroke volume (SV), which lead to an increased cardiac output (CO), are among the key hemodynamic changes. These are accompanied by a decrease in total systemic vascular resistance (SVR) and pulmonary vascular resistance, whereas total systemic vascular compliance increases. As a result, blood volume increases while blood pressure is relatively unchanged.^1^, ^2^, ^3^ The induced volume overload typical of pregnancy leads to changes in cardiac structure. This involves increased atrial and ventricular end‐diastolic volume, wall thickness, and mass, which constitute physiological remodeling.^1^, ^2^, ^3^, ^4^, ^5^, ^6^, ^7^

When maternal adaptations are insufficient, both maternal and fetal morbidities can arise. For example, maternal hypertension can result from an inadequate SVR reduction.^3^, ^8^ Moreover, cardiac remodeling is found to be eccentric, as in athletes, when physiological pregnancies occur, whereas concentric hypertrophy, as typical of some cardiovascular diseases, for example, valve diseases, is often found in pathological pregnancies, for example, preeclamptic women.^1^, ^8^ Thus, the analysis of both hemodynamic and geometrical changes is fundamental to developing a full understanding of both normal and high‐risk pregnancies.

Despite the fundamental role of the maternal cardiovascular system in pregnancy, prior modeling studies have focused almost exclusively on the fetal side or on the interaction between mother and fetus,^9^, ^10^, ^11^, ^12^, ^13^ and as far as we are aware, there are only two works that study the whole maternal blood circulation during pregnancy.^14^, ^15^ Corsini et al. were the first to describe a lumped parameter model of the maternal circulation, achieving reasonable agreement between model outputs and in vivo data.^14^ More recently, Carson et al. described a one‐dimensional model suitable for estimating volumetric blood flow to the uterus via the utero‐ovarian communicating arteries and for capturing wave propagation phenomena in the utero‐ovarian circulation.^15^ However, although these focused on the mother, they only replicated the hemodynamic changes but did not consider the structural remodeling that occurs during gestation. The difficulty of including structural changes probably arises from the lack of a complete and in‐depth knowledge of the complex mechanisms that trigger the typical alterations of pregnancy.^16^ Indeed, to date, molecular details of cardiac remodeling are not fully understood but it is thought that cardiac remodeling is a complex process driven by (1) wall stress homeostasis, (2) evolving hemodynamics, and (3) hormonal signaling.^16^ Thus, in the present work, we analyzed both the hemodynamic and the cardiac alterations typical of pregnancy for the first time. Regarding the cardiac remodeling, we investigated to what extent a relatively simple algorithm involving the first two of the previously stated factors could predict typical cardiac remodeling during a normal pregnancy. We, therefore, represented cardiac remodeling by following the work of Maksuti et al.,^17^ in which myofiber stress ($\sigma_{f}$) and chamber wall shear stress ($\sigma_{\mathrm{wss}}$) homeostasis principles are assumed to drive cardiac remodeling. Therefore, from the viewpoint of the heart, cardiac stresses are considered to be the “hemodynamic stimuli” in a phenomenological sense, neglecting at this stage precise mechanisms. To investigate cardiac remodeling in pregnancy, we hence implemented two lumped parameter models of the whole maternal circulation, one with a controlled remodeling algorithm (i.e., imposed homeostatic stresses' values) and the other with no controlled remodeling algorithm (i.e., stresses are free to vary, and any changes in cardiac geometry are caused by altered loading conditions). The results of the two models were compared with clinical data in the context of normal pregnancy.

## 2 METHODS

A lumped parameter modeling approach was chosen because it allows fast simulations and adequately captures the major features of cardiac function and vascular pressure and flow dynamics throughout the whole cardiovascular system, which is desirable for investigating adaptive remodeling processes. The methodology provides for the division of the system into compartments whose number depends on the level of accuracy required.

To study and analyze the hemodynamic and structural changes during pregnancy, we first considered the circulation of the non‐pregnant case (NPC). We then represented typical changes for each trimester, considering: (i) first (T1), (ii) second (T2), and (iii) third (T3) trimesters, and (iv) the end of pregnancy (Term). Note that, for our purposes, we coupled a hemodynamic model with cardiac remodeling algorithms. The latter are explained in the corresponding sections.

### 2.1 Hemodynamic model

**FIGURE 1:**
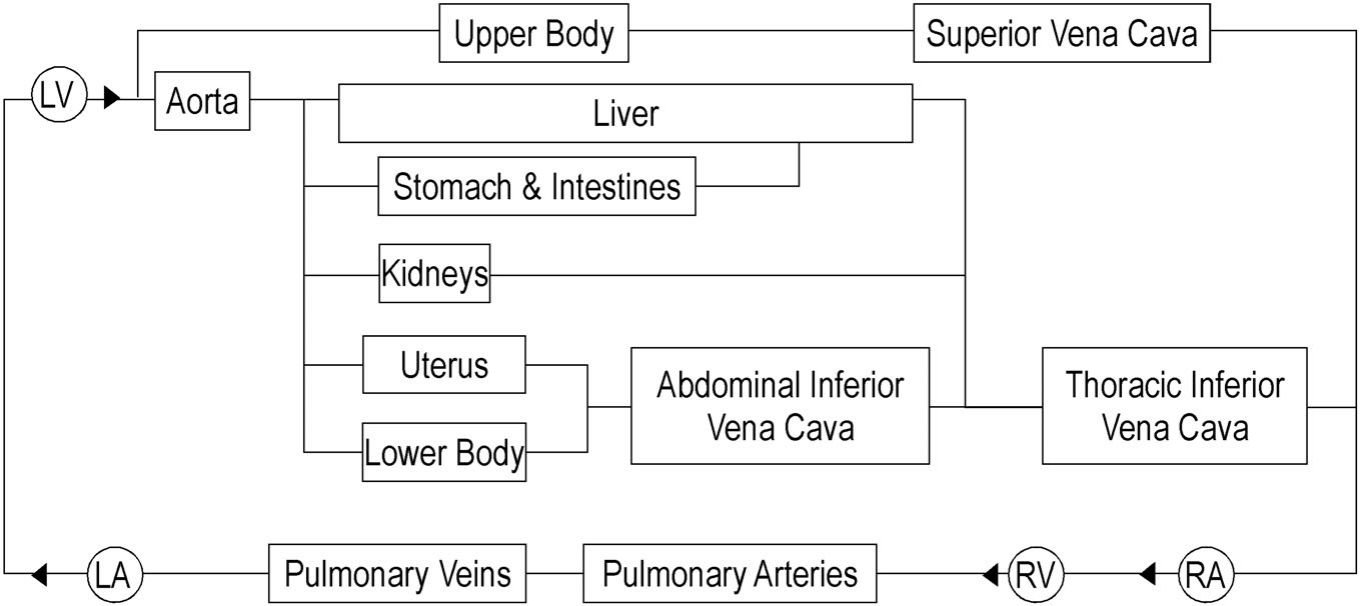
Circulation model composed of: left ventricle (LV), aorta, liver, stomach and intestines, kidneys, uterus, upper and lower body, great veins (superior and thoracic and abdominal inferior vena cava), right ventricle (RV), right atrium (RA), pulmonary arteries and veins, left atrium (LA), and heart valves (►)

The model builds on work presented in Comunale et al.^18^ in which a lumped circulation model was parameterized specifically for simulating hemodynamics in a representative woman (see Data S1 for details). This comprised systemic and pulmonary circulations, the four heart chambers, and valves (Figure 1). To simulate the great vessels, we reproduced the resistance to flow due to blood viscosity (*R*), the compliance of the vessel (*C*), and inertial effects (*L*) (Figure S1). The systemic organs were represented by considering the arterial and venous resistive and compliance effects ($R_{\mathrm{art}},C_{\mathrm{art}},R_{\mathrm{ven}},$ and $C_{\mathrm{ven}}$), as well as microvascular bed resistance ($R_{\mathrm{vb}}$) (Figure S1). The heart was modeled via the well‐recognized myocardial elastance theory.^19^ Myocardial activity was represented by means of a time‐varying elastance ($E\left(t\right)$) as in Mynard and Smolich,^20^ considering the elastance variations during contraction ($g_{1}$) and relaxation ($g_{2}$) as

(1) $$E\left(t\right)=k\left(\frac{g_{1}}{1+g_{1}}\right)\left(\frac{1}{1+g_{2}}\right)+E_{\min},$$

and $g_{1}={\left(\frac{t-t_{\text{onset}}}{\tau_{1}}\right)}^{m_{1}},\;g_{2}={\left(\frac{t-t_{\text{onset}}}{\tau_{2}}\right)}^{m_{2}}$here k is a scaling factor to guarantee that $\max\left(E\left(t\right)\right)=E_{\max}$, being $E_{\max}$ and $E_{\min}$ the maximum and minimum chamber elastance, respectively. $\tau_{1}$ and $\tau_{2}$ determine the timing of contraction and relaxation, $t_{\text{onset}}$ is a time‐shift for atrial contraction, and m controls the steepness. Equation (1) allows computation of pressure within each chamber ($p\left(t\right)$) as

(2) $$p\left(t\right)=E\left(t)(V\left(t\right)-V_{p=0}\right),$$

where $V\left(t\right)$ and $V_{p=0}$ are the chamber volume and the unstressed volume at zero pressure, respectively. Finally, pressure‐flow relations for heart valves were represented by the Bernoulli equation, while valve opening and closing dynamics were taken into account by considering the transvalvular pressure gradient, as in Mynard et al.^21^

### 2.2 Geometrical model

Cardiac remodeling is usually assessed by evaluating the radius and thickness of heart chambers. Thus, to study cardiac remodeling, we assigned a geometry to each heart chamber. Particularly, we assumed the geometry proposed in Maksuti et al.,^17^ where the left and right atria (LA, RA) were considered spheres, the left ventricle (LV) was a half ellipsoid, and the right ventricle (RV) was represented as a quarter ellipsoid (Figure S3). Each chamber was described by a wall thickness (*h*) and an inner radius (*r*), and the ventricular longitudinal dimension was defined as 3*r* (see Data S1 for the mathematical description). Interactions between chambers were neglected.

### 2.3 Pregnancy

Pregnancy was simulated in the hemodynamic model by applying the following alterations that reflect adaptive processes in the cardiovascular system during gestation. These adjustments reflect the altered hormonal levels, renin‐angiotensin‐aldosterone system (RAAS), and nervous system (e.g., increased sympathetic activities) which are fundamental to ensure the development of healthy pregnancies.^3^, ^22^, ^23^ Note that the focus of this present study is cardiac remodeling in pregnancy, and therefore we first imposed (rather than predicted) other adaptations as outlined below, and then we considered the cardiac alterations by coupling the remodeling algorithms.

2.3.1

*Blood flow distribution*. It is known that vascular resistances decrease during pregnancy.^1^, ^24^ Particularly, the distribution of CO to various organs changes according to trimester.^14^, ^25^, ^26^, ^27^ The blood distribution to each organ was imposed according to literature data (Table 1). Note that this determined a change in the values of the lumped parameters that describe each compartment, which allowed reproducing the different organs' perfusion typical of pregnancy (e.g., increased uterus perfusion) (see *Parameterization*).

**TABLE 1:** Blood flow distribution during gestation

% CO	NPC (%)	T1 (%)	T2 (%)	T3 (%)	Term (%)
Qliver	6.56	5.64	5.45	5.02	4.73
Qsi	26	32.36	34.55	34.98	34.27
Qub	22	18.91	18.27	16.84	15.85
Qk	21	24	23	21	18
Qut	0.44	4.17	5.60	10.88	15
Qlb	24	14.92	13.13	11.28	12.15

Abbreviation: NPC, non‐pregnant case, T1, first trimester, T2, second trimester, T3, third trimester, and Term, end of pregnancy. Qliver, hepatic flow, Qsi, flow to the stomach and intestines, Qub, upper body flow, Qk, renal flow, Qut, uterine flow, Qlb, lower body flow.

2.3.2

*Total vascular resistance and systemic global compliance*. SVR and systemic compliances were reduced and increased, respectively, according to data reported by Melchiorre et al.^1^ (Table S1).

2.3.3

*Heart rate*. Heart rate increases as pregnancy proceeds and changes reported in the literature^28^ were imposed as a model input (Table 2).

**TABLE 2:** Input parameters used to simulate the hemodynamics of pregnancy^28^

Parameters	NPC	T1	T2	T3	Term
Weight (kg)	56	62	65	70	75
Height (cm)	167	165	165	165	166
HR (bpm)	71	75	76	82	79
CO (L/min)	4.9	5.7	5.9	6.4	6.8
Perfusion pressure (mmHg)	76	70	72	76	76

Abbreviations: BSA, body surface area; CO, cardiac output; HR, heart rate.

2.3.4

*Heart valves*. Valve size increases during pregnancy,^29^, ^30^, ^31^, ^32^ however, little quantitative data are available.^31^, ^32^ Campos^31^ reported increases in the diameter of valves diameter, whereas Robson et al.^32^ reported changes in the valve area. To avoid the introduction of errors due to valve geometry assumptions, we imposed percentage area increases based on the values in Robson et al.^32^ Note that, since data for the tricuspid valve are not available, we assumed that the tricuspid valve increases in parallel with the pulmonary valve, as the mitral valve increases in relation to the aortic valve (i.e., %tricuspid valve area=%mitral valve area·%pulmonary valve area/%aortic valve area).

### 2.4 Cardiac remodeling

#### 2.4.1 Theoretical considerations

Cardiac remodeling leads to changes in mass, size, geometry, and function to preserve optimal hemodynamics.^33^ Since pregnancy is a condition of volume overload, it is known that the increased demands on the heart drives a reversible eccentric hypertrophy.^6^, ^7^, ^34^ However, the signaling pathways of gestational cardiac remodeling are not completely understood yet.^16^

Maksuti et al.^17^ proposed an algorithm to study cardiac remodeling in heart valve diseases. As a first approximation, they supposed that the main driving rule of cardiac remodeling is the preservation of myofiber stress and wall shear stress. Similar to the remodeling of blood vessels, the former has a crucial role in determining a variation in wall thickness; whereas the latter causes chamber dilation in response to increasing flow. These hypotheses derive from the numerous in vitro studies that report the transducer role of vascular endothelial cells, that is, these cells can sense and transduce biomechanical stimuli as myofiber and wall shear stresses, controlling vascular remodeling.^35^ Moreover, it has been suggested that the responses to shear forces that shape the developing heart can also contribute to abnormalities and diseases processes in the adult life.^35^ The human heart is known to have several types of endothelial cells,^36^, ^37^, ^38^ and it has been demonstrated that cardiac endothelial cells “*play an obligatory role in regulating and maintaining cardiac function*.”^37^ In addition, it is thought that the sensor role of endocardial endothelial cells are modulators of ventricular cardiomyocyte contractile function.^37^, ^38^ Thus, it is plausible to think that the endocardial endothelial cells may play a role in sensing and transducing wall shear stresses, and consequently, it is feasible that these cells contribute to the cardiac remodeling as a response to biomechanical stimuli.

To study cardiac remodeling in pregnancy, we hence started from the algorithm of Maksuti et al.^17^ and we updated it to consider the alterations typical of pregnancy. Indeed, although the key principles regarding myofiber and wall shear stresses were maintained, we added an additional aspect to the algorithm that accounts for the fact that remodeling during pregnancy differs from the pathological one because it is mainly directed toward supporting an increased CO. We, therefore, maintained the rules in which myofiber stress and wall shear stress are kept constant (equal to NPC values) and we added a rule that determines a change in blood volume to meet the required CO. This can be seen as an increase in the total blood volume within the body and a variation in myofiber length that, according to the Frank–Starling law, determines a change in myocardial contractility/relaxation. Indeed, to satisfy the required hemodynamics during pregnancy, blood volume increases and so does the size of the chambers. Note that, since the cardiac remodeling algorithm was coupled to the hemodynamic model, we simulated both the vascular tree adaptation (by means of the hemodynamic model), and the structural alterations (considering the remodeling algorithm) typical of pregnancy. This is a key point since during pregnancy both the modifications are important to ensure the development of healthy gestations and usually in numerical models only the hemodynamic variations are reproduced.^14^, ^15^

Considering the remodeling algorithm, we further updated the algorithm of Maksuti et al.^17^ For the ventricular wall stress, differently from atria and from Maksuti et al.,^17^ we used the clinical formula:

(3) $$\sigma_{f}=P_{\mathrm{es}}∙\frac{2r}{4\cdot PW_{s}\cdot \left(1+\frac{PW_{s}}{2r}\right)},$$

where $P_{\mathrm{es}}$, $PW_{s}$, and r are the ventricular pressure, thickness, and radius at end‐systole, respectively.^8^, ^28^, ^39^ This formula allows us to compare the computed $\sigma_{f}$ with clinical data, but also to evaluate the ability of the myocardium to adapt to the volume overload condition. Indeed, it has been shown that the myocardium adapts to the overload condition by maintaining normal end‐systolic stress values, both in pathological conditions^40^, ^41^ and in athletes performing isotonic exercise,^42^ the latter being a state comparable to pregnancy. Moreover, the end‐systolic stress is thought to best represent the afterload that limits ejection (compared to the mean stress), that is, it is considered the marker that limits the ejection, because it represents the moment at which the ejection ends because the myocardium has reached the maximum force.^43^ (Note that Equation (3) derives from the work of Grossman et al.^39^ in which stresses are given in 10^3^ dyne/cm^2^. However, in Figure 3 of the cited work,^39^ there is a typographical error and the stresses are reported in g/cm^2^, which does not represent a stress unit. This error has been propagated and, in clinical works, the formula has been updated with a conversion factor of 1.35 to convert mmHg to g/cm^2^,^44^ resulting in values that if converted to the proper unit do not agree with physiological values of pressures, radii, and thicknesses.^8^, ^28^ Thus, we here considered the initial formula without the conversion factor and the results are reported in mmHg which is more familiar to clinicians than 10^3^ dyne/cm^2^.).

We also updated the formula used for the atria by considering the generalized law of Laplace specifically derived for spheres^45^:

(4) $$\sigma_{f}=\frac{P\cdot r}{2h}.$$

In this case, due to lack of specific knowledge on atrial stress, we maintained the rule of Maksuti et al.^17^ and we considered the mean value over the heartbeat. Note that, by applying specific heart chamber's formulas, we updated the algorithm of Maksuti et al., which imposed $\sigma_{f}=p∙\frac{3}{\ln\left(1+\frac{v_{\text{wall}}}{v_{\text{lumen}}}\right)}$ ^17^ for all the heart chambers.

**FIGURE 2:**
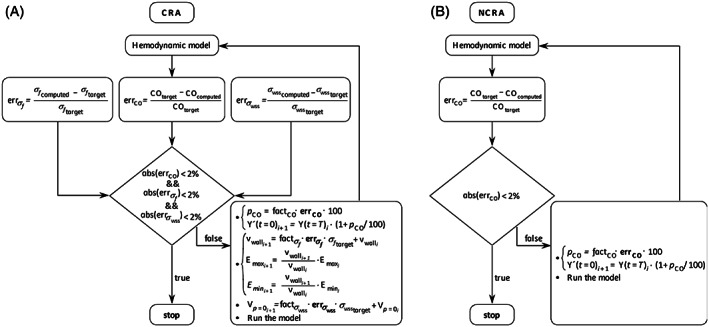
Schematic representations of (A) the controlled remodeling algorithm (CRA) and (B) the no‐controlled remodeling algorithm (NCRA). ${\mathrm{err}}_{\sigma_{f}}$ is the error between the computed (${\sigma_{f}}_{\text{computed}}$) and the desired myofiber stress (${\sigma_{f}}_{\text{target}}$), ${\mathrm{err}}_{\mathrm{CO}}$ is the error between the computed ($CO_{\text{computed}}$) and the desired cardiac output ($CO_{\text{target}}$), and ${\mathrm{err}}_{\sigma_{\mathrm{wss}}}$ is the error between the computed (${\sigma_{\mathrm{wss}}}_{\text{computed}}$) and the desired wall shear stress (${\sigma_{\mathrm{wss}}}_{\text{target}}$). If ($\mathrm{abs}\left({\mathrm{err}}_{\mathrm{CO}}\right)<2\%$ && $\mathrm{abs}\left({\mathrm{err}}_{\sigma_{f}}\right)<2\%$ && $\mathrm{abs}\left({\mathrm{err}}_{\sigma_{\mathrm{wss}}}\right)<2\%$) is false, the following changes were applied: (i) variations to the initial values of the model's variables ($Y^{\prime }{\left(t=0\right)}_{i+1}$), (ii) adjustments of wall chamber volumes ($v_{\mathrm{wal}l_{i+1}}$), and maximum and minimum elastances (${E_{\max}}_{i+1}$ and ${E_{\min}}_{i+1}$), and (iii) changes of unstressed chamber volumes $V_{p=0_{i+1}}$. (i) was obtained by computing and imposing the percentage change ($p_{\mathrm{CO}}$), with $\mathrm{fac}t_{\mathrm{CO}}=0.01$, and $Y^{\prime }{\left(t=0\right)}_{i+1}$ the new initial value computed from the last value of the previous heartbeat simulated ($Y{\left(t=T\right)}_{i}$). (ii) determines the variations of wall chamber volumes and elastances from the previous iteration values ($v_{\mathrm{wal}l_{i}}$, ${E_{\max}}_{i}$ and ${E_{\min}}_{i}$) with $\mathrm{fac}t_{\sigma_{f}}=0.005$. (iii) computes the unstressed volumes from the previous values $V_{p=0_{i}}$ and $\mathrm{fac}t_{\sigma_{\mathrm{wss}}}=1000$. Please see the section “Cardiac remodeling” for a detailed description of the two models

Complete details on the remodeling algorithm are reported in the next subsection “Algorithm,” whereas for a schematic representation of the coupling between the hemodynamic model and the remodeling algorithms please see Figure 2. Note that, in the following, controlled remodeling algorithm (CRA) stands for the hemodynamic model coupled with the controlled remodeling algorithm, whereas no controlled remodeling algorithm (NCRA) refers to the hemodynamic model coupled with no controlled remodeling algorithm.

#### 2.4.2 Algorithm

Cardiac remodeling during pregnancy was modeled via three rules: (i) the increase in blood volume to maintain target CO, (ii) the maintenance of physiological myofiber stress, and (iii) the maintenance of physiological wall shear stress.

The above three rules were implemented as described in the following.

- *Increase in blood volume*: this was realized by changing the initial values of the model's variables so that the difference between the computed ($CO_{\text{computed}}$) and the desired cardiac output ($CO_{\text{target}}$) met a given tolerance. In fact, in lumped parameter models the amount of blood in the circuit is determined by the initial conditions. The target value for each trimester was taken from Melchiorre et al.^28^ (Table 2) and a tolerance of 2% was imposed. In particular, first, the error (${\mathrm{err}}_{\mathrm{CO}}$) was computed as
(5) $${\mathrm{err}}_{\mathrm{CO}}=\frac{CO_{\text{target}}-CO_{\text{computed}}}{CO_{\text{target}}},$$
  then, if $\mathrm{abs}\left({\mathrm{err}}_{\mathrm{CO}}\right)>2\%$, variations were applied to the initial values of the model's variables by computing and imposing the percentage change ($p_{\mathrm{CO}}$) in the following way
(6) $$\left\{\begin{array}{c} p_{\mathrm{CO}}={\text{fact}}_{\mathrm{CO}}\cdot {\mathrm{err}}_{\mathrm{CO}}\cdot 100\\ Y\prime {\left(t=0\right)}_{i+1}=Y{\left(t=T\right)}_{i}\cdot \left(1+p_{\mathrm{CO}}/100\right) \end{array}\right.$$
  where fact_CO_ = 0.01 and is factor to reduce the variations imposed to the initial values to guarantee stability, that is, avoiding big fluctuations in the system, and $Y^{\prime }{\left(t=0\right)}_{i+1}$ is the new initial value computed from the last value of the previous heartbeat simulated ($Y{\left(t=T\right)}_{i}$). The use of $Y{\left(t=T\right)}_{i}$ allowed to work in the range of stability of the system, reducing the iterations required to converge. Note also that $p_{\mathrm{CO}}$ can be positive or negative depending on $CO_{\text{computed}}$ and hence allowing both higher and smaller initial values.
- *Maintenance of physiological myofiber stress*: similar to the work of Maksuti et al.,^17^ this rule is realized by changing myocardial wall volume, and assuming that the myocardium is distributed among the four heart chambers in proportion to their stiffness and contractility.^17^ The target value (during pregnancy) for each heart chamber is the NPC value reported in Table S2 and, as previously, a tolerance of 2% was imposed. Thus, first, the error (${\mathrm{err}}_{\sigma_{f}}$) between the computed (${\sigma_{f}}_{\text{computed}}$) and the desired myofiber stress (${\sigma_{f}}_{\text{target}}$) was computed as
(7) $${\mathrm{err}}_{\sigma_{f}}=\frac{{\sigma_{f}}_{\text{computed}}-{\sigma_{f}}_{\text{target}}}{{\sigma_{f}}_{\text{target}}}.$$
  Then, if $\mathrm{abs}\left({\mathrm{err}}_{\sigma_{f}}\right)>2\%$ the following changes were applied
(8) $$\left\{\begin{array}{c} v_{\mathrm{wal}l_{i+1}}=\mathrm{fac}t_{\sigma_{f}}∙\mathrm{er}r_{\sigma_{f}}∙{\sigma_{f}}_{\text{target}}+v_{\mathrm{wal}l_{i}}\\ \begin{array}{c} {E_{\max}}_{i+1}=\frac{v_{\mathrm{wal}l_{i+1}}}{v_{\mathrm{wal}l_{i}}}∙{E_{\max}}_{i}\\ {E_{\min}}_{i+1}=\frac{v_{\mathrm{wal}l_{i+1}}}{v_{\mathrm{wal}l_{i}}}∙{E_{\min}}_{i} \end{array}\; \end{array}\right.,$$
  where ${\mathrm{fact}}_{\sigma f}=0.005$ and has the same role of $\mathrm{fac}t_{\mathrm{CO}}$, $\;v_{\mathrm{wal}l_{i+1}}$ is the new value of wall chamber computed from the previous iteration value ($v_{\mathrm{wal}l_{i}}$), and ${E_{\max}}_{i+1}$ and ${E_{\min}}_{i+1}$ are the new maximum and minimum heart elastance (i.e., maximum force and stiffness, respectively) computed from the previous iteration values (${E_{\max}}_{i}$ and ${E_{\min}}_{i}$), respectively. Note also that $v_{\text{wall}}$, $E_{\max}$, and $E_{\min}$ are heart chamber‐dependent. Notice that, differently from Maksuti et al.,^17^ for the ventricles we used the clinical formula (Equation (3)).^8^, ^28^ The derivation of Equation (3) is well explained in Grossman et al.^39^ and it is here reported for completeness. Ventricular stress depends on chamber geometry and pressure. It has been shown that for ellipsoidal and spherical shapes, there is an average stress ($\sigma_{m}$, i.e., the force per unit area) that acts at the midplane to the heart in the direction of the apex to base length. The formula can be derived by imposing the equilibrium between the meridional wall forces $\left(\sigma_{m}\cdot \pi \left(R_{o}^{2}-R_{i}^{2}\right)\right)$ and the pressure loading ($\mathrm{P\pi}R_{i}^{2}$). Thus,
(9) $$\sigma_{m}\cdot \pi \left(R_{o}^{2}-R_{i}^{2}\right)=\mathrm{P\pi}R_{i}^{2},$$
  where $R_{o}$ is the outer radius of the chamber and $R_{i}$ is the inner radius, thus $\left(R_{o}-R_{i}\right)=h$, that is, the wall thickness. By properly rearranging and changing the notations, Equation (9) becomes Equation (3). On the other hand, for the atria, we preferred to use the generalized law of Laplace, which takes into account the imposed spherical shape (Equation (4)).^45^ Notice that, Equation (4) is applicable for atria since, for both RA and LA, during pregnancy the ratio h/r varies in the range 0.03−0.06 which satisfied the required condition of thin‐walled spheres, that is, h/r<.1.
- *Maintenance of physiological wall shear stress*: analogous to the previous rule and as previously reported, this rule was implemented in a similar way to that described by Maksuti et al.^17^ In particular, the maintenance of $\sigma_{\mathrm{wss}}$ was obtained by varying the unstressed volume ($V_{p=0}$) of Equation (2). This comes from vessel's remodeling in which it is known that wall shear stress affects the arterial diameter.^45^ Since $V_{p=0}$ represents the heart chambers' unstressed volume, altering this parameter is equivalent to alter the chambers' dimensions, thus reflecting the effect of $\sigma_{\mathrm{wss}}$. The target value (during pregnancy) for each heart chamber was, again, the NPC value reported in Table S2 and, as previously, a tolerance of 2% was imposed. Thus, in analogy to the previous two rules, first, the error (${\mathrm{err}}_{\sigma_{\mathrm{wss}}}$) between the computed (${\sigma_{\mathrm{wss}}}_{\text{computed}}$) and the desired wall shear stress (${\sigma_{\mathrm{wss}}}_{\text{target}}$) was computed as
(10) $${\mathrm{err}}_{\sigma_{\mathrm{wss}}}=\frac{{\sigma_{\mathrm{wss}}}_{\text{computed}}-{\sigma_{\mathrm{wss}}}_{\text{target}}}{{\sigma_{\mathrm{wss}}}_{\text{target}}}.$$
  Then, if $\mathrm{abs}\left({\mathrm{err}}_{\sigma_{\mathrm{wss}}}\right)>2\%$, $V_{p=0}$ was adjusted as
(11) $$V_{p=0_{i+1}}=\mathrm{fac}t_{\sigma_{\mathrm{wss}}}∙\mathrm{er}r_{\sigma_{\mathrm{wss}}}∙{\sigma_{\mathrm{wss}}}_{\text{target}}+V_{p=0_{i}}$$
  where $\mathrm{fac}t_{\sigma_{\mathrm{wss}}}=1000$ and has the same role of $\mathrm{fac}t_{\mathrm{CO}}$ and $\mathrm{fac}t_{\sigma_{f}}$. Also, $V_{p=0}$ is heart chamber‐dependent. Moreover, $\sigma_{\mathrm{wss}}$ was computed assuming a laminar flow of Poiseuille's type through a cylindrical pipe as
(12) $$\left\{\begin{array}{c} \sigma_{\mathrm{wss}}=\frac{4\mu q_{\text{chamber}}}{\pi r^{3}}\;\\ q_{\text{chamber}}=\frac{\left|q_{\text{inlet}}\right|+|q_{\text{outlet}}|}{2} \end{array}\right.,$$
  where μ is the dynamic viscosity, and $q_{\text{inlet}}$ and $q_{\text{outlet}}$ are the inlet and outlet valve blood flow, respectively. Note that expression of Equation (12) was used as a first approximation and it does not strictly apply to the geometries considered or if flow conditions are turbulent.

Finally, the two lumped parameter models were hence built as follow:

1. *CRA*: Results from the set of the three conditions described above. CRA was hence implemented in the woman‐specific model to describe the pregnancy in terms of both the hemodynamic variations and the cardiac remodeling conditioned by homeostatic values of stresses.
2. *NCRA* considers only the hemodynamic changes of the pregnancy whereas the cardiac remodeling was unconditional, that is, NCRA solves only rule (i).

Note that, we always referred to $\sigma_{f}$ and $\sigma_{\mathrm{wss}}$ of the LV due to the absence of references for the other chambers. In fact, only for LV in vivo data were available.^8^, ^17^, ^28^, ^46^

### 2.5 Parameterization

To properly calibrate the hemodynamic model, we used the methodology proposed in Comunale et al.,^18^ which requires a target CO, perfusion pressure (PerfP), that is, the mean pressure that perfuses the systemic organs, along with prescribed RC time constants for the systemic and pulmonary circulations, flow distribution among organs, and assumed ratio of arterial to venous compliance. Particularly, to account for published clinical data, values of resistances and compliances change at every trimester. As reported in Comunale et al.,^18^ the total resistance ($R_{\mathrm{tot}}=R_{\mathrm{art}}+R_{\mathrm{vb}}+R_{\mathrm{ven}}$) of each compartment is computed as $R_{\mathrm{tot}}=\text{PerfP}/\left(\%\mathrm{CO}∙\mathrm{CO}\right)$, divided in $5\%R_{\mathrm{tot}}$ to the arterial side, $92\%R_{\mathrm{tot}}$ to the vascular bed ($R_{\mathrm{vb}}$) and $3\%R_{\mathrm{tot}}$ to the venous side. The arterial compliance was then computed as $C_{\mathrm{art}}=\tau /R_{\mathrm{vb}}$, with τ the time constant of each circulation ($\tau_{\mathrm{sys}}=0.81\;s$ ^47^ and $\tau_{\mathrm{pul}}=0.5\;s,$ ^48^ assuming that they did not change during pregnancy); and finally, $C_{\mathrm{ven}}=30\cdot C_{\mathrm{art}}.$ ^49^ For the heart, parameters values of Equation (1) and (2) were taken from Mynard et al.^21^ and Mynard and Smolich.^20^ Note that, for the NPC simulation, adjustment of the unstressed volume ($V_{p=0}$) of Equation (2) was necessary in order to meet the desired female‐specific hemodynamics. The obtained values were then kept constant during the different phases of gestation. Note that the assumption of constant $\tau_{\mathrm{sys}}$ and $\tau_{\mathrm{pul}}$ throughout pregnancy is a limitation of the work. However, during pregnancy heart rate varied by only 15%.

### 2.6 Clinical variables

To run the model, we computed several clinically‐relevant variables. Here we report the mathematical description for those that need specification; see the Data S1 for a full description. (i) The ventricular myofiber stress that controls cardiac remodeling was computed using the clinical formula (see Equation (3)). (ii) Chamber mass (*M*) was computed as $M=\rho \cdot V_{\mathrm{myo}}$, with ρ the myocardial density (1.04 g/ml) and $V_{\mathrm{myo}}$ the myocardial volume. (iii) We assumed for the NPC case, a total blood volume ($V_{\mathrm{TOT}}$) comprising 70% unstressed volume ($V_{{\mathrm{TOT}}_{U}}$) and 30% stressed volume ($V_{{\mathrm{TOT}}_{S}}$).^50^, ^51^ $V_{{\mathrm{TOT}}_{S}}$ was directly computed from simulations as the sum of the different volume compartments considered in Figure 1, and given $V_{{\mathrm{TOT}}_{S}}$, $V_{\mathrm{TOT}}$, and $V_{{\mathrm{TOT}}_{U}}$ can then be derived from the assumed $V_{{\mathrm{TOT}}_{S}}/V_{{\mathrm{TOT}}_{U}}$ ratio. Experiments in animals suggest that during pregnancy the unstressed volume stays the same^52^ or increases by up to 33% at term.^53^ For humans, it is well accepted that $V_{{\mathrm{TOT}}_{U}}$ rises,^54^, ^55^, ^56^, ^57^ however, the amount of the increase is still unclear. For these reasons, during pregnancy, we evaluated the change in $V_{{\mathrm{TOT}}_{U}}$ and $V_{\mathrm{TOT}}$ assuming $V_{{\mathrm{TOT}}_{U}}$ increases between 0% and 33%.

### 2.7 Simulations

To analyze the different stages of pregnancy, we started by considering the female‐specific circulation of an “average” non‐pregnant woman, calibrating the model to meet the NPC hemodynamics. Particularly, we adopted the clinical data of Melchiorre et al.,^28^ that is, we simulated a woman of 56 kg and 167 cm, having a heart rate of 71 bpm, a CO of 4.9 L/min, and a perfusion pressure of 76 mmHg. The valve areas were defined in agreement to the work of Pettersen et al.,^58^ which linked the valve dimensions to the body surface area (BSA). The latter was computed by applying the Schlich formula that is specific for women and resulting in BSA = 1.56 m^2^. We also prescribed a total myocardial volume of 109 ml derived from Melchiorre et al.^28^ and assuming a myocardial distribution in proportion to the heart mechanical properties as suggested by Maksuti et al.^17^ We then considered the three trimesters of pregnancy (Table 2) by first imposing the hemodynamic variations and then considering the remodeling algorithms.

To identify the effect of $\sigma_{f}$ and $\sigma_{\mathrm{wss}}$ on hemodynamics, we also ran the remodeling algorithm with alternative $\sigma_{f}$ target values that were within physiological ranges (±20%).^8^, ^28^, ^46^ In regards to $\sigma_{\mathrm{wss}}$, since no specific clinical data were available, we chose to follow the approach of Maksuti et al.^17^ and to apply the same percentage variation for both $\sigma_{f}$ and $\sigma_{\mathrm{wss}}$.

We also performed a global sensitivity analysis to ascertain the influence of parameters on the model. To that aim, we followed the Monte Carlo‐based approach proposed by Saltelli^59^ and applied to cardiovascular 0D‐1D models by Zhang et al.^60^ We prescribed an uncertainty of 15% for all the inputs and analyzed the behavior of the sensitivity indices for some meaningful outputs.

The system of ODE equations was run by exploiting the built‐in MATLAB® function *ode15s*, solving a closed‐loop system for 30 cycles, allowing the system to converge. Results were obtained after reaching the periodic steady state.

## 3 RESULTS

**FIGURE 3:**
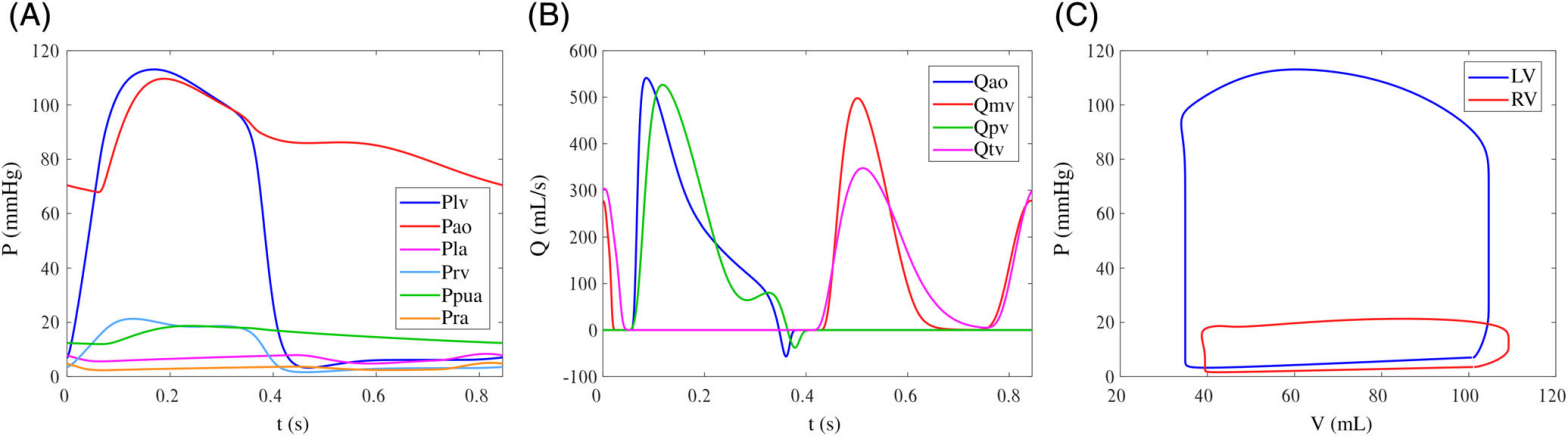
Hemodynamic outputs of the non‐pregnant case (NPC) model. (A) Pressures, (B) flows, and (C) pressure‐volume loops. LV, left ventricle; RV, right ventricle; LA, left atrium; and RA, right atrium. Plv, LV pressure; Pao, aortic pressure; Pla, LA pressure; Prv, RV pressure; Ppua, pulmonary arterial pressure; Pra, RA pressure; Qao, aortic valve flow; Qmv, mitral valve flow; Qpv, pulmonary valve flow; Qtv, tricuspid valve flow

The ability of the NPC model to represent female‐specific hemodynamics is first evaluated. Table 3 shows the global hemodynamic variables. There is a very good agreement between the characteristic female‐specific in vivo variables and the model outputs, with hemodynamic and chamber indices within the physiological ranges and close to the mean/median value of the reference. Physiological trends are also found when considering the waveforms. Figure 3 represents the pressures, flows, and volumes, and these well resemble the characteristic physiological hemodynamics. The LV is characterized by higher pressure and lower volume compared to the RV with the same SV of about 70 ml and a pressure ratio of about 6:1. Moreover, the valve model replicates the small amount of backward flow through the semilunar valves and the typical double peaks shape of the atrioventricular valve flow.^62^, ^63^, ^64^, ^65^, ^66^

**TABLE 3:** Outputs of the non‐pregnant case (NPC) simulations

Parameters	NPC	Reference
SVR (dynes·s/cm^5^)	1327	1278 (1133–1496)^28^
SBP (mmHg)	110	110 (100–115)^28^
DBP (mmHg)	68	70 (60–80)^28^
MAP (mmHg)	82	83 (71–90)^28^
CO (L/min)	4.9	4.9 (4.3–5.8)^28^
LVEDV (ml)	105	96 ± 23 (52–141)^61^
LVESV (ml)	34	32 ± 9 (13–51)^61^
RVEDV (ml)	109	106 ± 24 (58–154)^61^
RVESV (ml)	39	40 ± 14 (12–68)^61^
SV (ml)	69	70 (66–79)^28^
EF (%)	66	65 (55–69)^28^
CW (mmHg L per min)	403	407 (333–478)^28^
LVM (g)	88	88 (71–110)^28^
RWT (−)	0.29	0.32 (0.27–0.36)^28^
LAD (cm)	4.5	3.1 (2.8–3.3)^28^
LVEDD (cm)	5.0	4.4 (4.2–4.7)^28^

*Note*: The values are compared with female‐specific in vivo data reported as median (interquartile range) or mean ± SD with 95% confidence intervals (1.96 SD) in parentheses.

Abbreviations: $\sigma_{f},$ the myofiber stress; CO, cardiac output; CW, cardiac work (CW=CO·MAP); DBP, diastolic blood pressure; EF, ejection fraction; MAP, mean arterial pressure; LAD, left atrial diameter; LVEDD, left ventricular end‐diastolic diameter; LVEDV, left ventricular end‐diastolic volume; LVESV, left ventricular end‐systolic volume; LVM, left ventricular mass; NPC, non‐pregnant case; RVEDV, right ventricular end‐diastolic volume; RVESV, right ventricular end‐systolic volume; RWT, relative wall thickness; SBP, systolic blood pressure; SV, stroke volume; SVR, total systemic vascular resistance.

**FIGURE 4:**
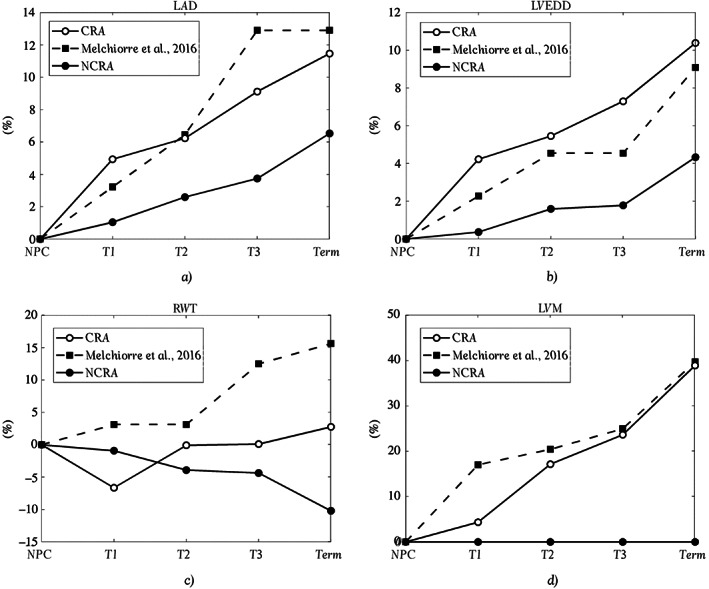
Percentage changes in the outputs of the simulations at the different trimesters of pregnancy from the non‐pregnant case (NPC) value. The results are compared with in vivo data.^28^ (A) Mean left atrial diameter, (B) left ventricular end‐diastolic diameter, (C) relative wall thickness, and (D) left ventricular mass. Black open dotted line, the controlled remodeling algorithm (CRA) simulation, black filled dotted line, the results with no controlled remodeling algorithm (NCRA), and in black squared dashed line, the in vivo data^28^

Table 4 shows the global hemodynamic variables during pregnancy, distinguishing the outputs between NCRA and CRA. The two models return almost the same overall hemodynamics in terms of pressures and CO; however, as the pregnancy proceeds, the differences in chamber volumes, performance, and geometrical indices between the reference values and the NCRA outputs increase. Figure 4 shows the percentage variations from NPC for the mean atrial diameter (LAD), the end‐diastolic LV diameter (LVEDD), the relative wall thickness (RWT) (i.e., $\mathrm{RWT}=2\cdot h_{\text{LVED}}/\text{LVEDD}$, where $h_{\text{LVED}}$ is the LV thickness at the end of diastole), and the LV mass (LVM). As pregnancy progresses, the diameters increase slightly or are approximately constant for NCRA (maximum increment of about 6%), but increase for CRA. The latter resulting in good agreement with the values of Melchiorre et al.^28^ with the maximum difference being for LAD at T2 of about 4%. With regard to RWT, both the models return smaller values compared to the reference values, which increase slightly as pregnancy progresses. RWT slightly decreases for NCRA, whereas it is approximately constant or increases very slightly for CRA. Finally, LV mass computed with CRA agrees very well with Melchiorre et al.^28^ with an NPC value of 88 g that increases during pregnancy until a final value of 122 g, equal to a 40% increment. For this configuration, the greatest difference between computed and clinical values is found at T1 with ~92 and 103 g, respectively. On the other hand, NCRA naturally returns a constant value of 88 g due to the absence of the remodeling algorithm.

**TABLE 4:** Comparison of pregnancy simulations with no controlled remodeling algorithm (NCRA) and with the controlled remodeling algorithm (CRA) against in vivo data^28^

Parameters	T1			T2		
	Model		Reference	Model		Reference
	NCRA	CRA		NCRA	CRA	
SVR (dynes/s per cm^5^)	1091	1096	1059 (936–1234)	1121	1129	1093 (863–1248)
SBP (mmHg)	101	103	100 (90–106)	106	110	100 (98–110)
DBP (mmHg)	64	66	63 (60–70)	69	70	68 (60–72)
MAP (mmHg)	76	78	77 (70–83)	81	83	79 (73–83)
CO (L/min)	5.6	5.7	5.7 (5.1–6.5)	5.8	5.9	5.9 (5.0–7.3)
LVEDV (ml)	106	118	—	110	121	—
LVESV (ml)	30	41	—	33	42	—
SV (ml)	75	76	76 (66–87)	76	78	78 (67–93)
EF (%)	70	65	61 (56–66)	69	64	63 (55–67)
CW (mm Hg L per min)	426	445	445 (383–513)	469	491	469 (391–576)
LVM (g)	88	92	103 (83–127)	88	103	106 (92–127)
RWT (−)	0.29	0.27	0.33 (0.30–0.37)	0.28	0.29	0.33 (0.29–0.37)
LAD (cm)	4.6	4.8	3.2 (2.9–3.4)	4.7	4.8	3.3 (3.0–3.6)
LVEDD (cm)	5.1	5.3	4.5 (4.3–4.8)	5.1	5.3	4.6 (4.4–4.8)

Parameters	T3			Term		
	Model		Reference	Model		Reference
	NCRA	CRA		NCRA	CRA	
SVR (dynes/s per cm^5^)	991	1000	977 (828–1177)	1017	1035	1000 (832–1138)
SBP (mmHg)	105	107	100 (98–110)	112	118	110 (100–120)
DBP (mmHg)	67	67	70 (60–72)	71	73	70 (60–75)
MAP (mmHg)	79	80	83 (73–87)	85	88	83 (74–90)
CO (L/min)	6.4	6.4	6.4 (5.4–7.8)	6.7	6.8	6.8 (6.0–7.7)
EDV (ml)	111	127	—	119	137	—
ESV (ml)	31	48	—	33	50	—
SV (ml)	78	78	80 (69–97)	84	86	83 (76–95)
EF (%)	71	61	60 (54–65)	71	63	60 (55–64)
CW (mmHg L per min)	509	512	524 (437–607)	565	598	564 (475–649)
LVM (g)	88	109	110 (88–130)	88	122	123 (104–143)
RWT (−)	0.28	0.29	0.36 (0.31–0.43)	0.26	0.30	0.37 (0.31–0.38)
LAD (cm)	4.7	5.0	3.5 (3.3–3.8)	4.8	5.1	3.5 (3.2–3.7)
LVEDD (cm)	5.1	5.4	4.6 (4.4–5.0)	5.3	5.6	4.8 (4.4–4.9)

*Note*: The clinical data are reported as median (interquartile range). See Table 3 for abbreviation.

**FIGURE 5:**
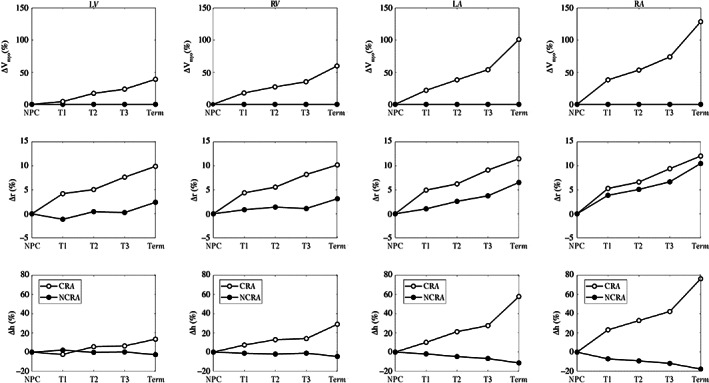
The percentage of changes in the outputs of the simulations for the four heart chambers at the different trimesters of pregnancy. In the first row, the myocardial volume, in the second row, the mean chamber's radius, and in the third row, the mean chamber's thickness. Black open dotted line, the controlled remodeling algorithm (CRA) simulation, black filled dotted line, the results with no controlled remodeling algorithm (NCRA)

Figure 5 reports the percentage variations of the geometrical indices for each heart chamber. As expected, NCRA predicts a constant myocardial volume during pregnancy (first row). CRA instead captures the increase in $V_{\mathrm{myo}}$ with a percentage increment at Term of about 40%, 60%, 100%, and 130% for LV, RV, LA, and RA, respectively. For the mean chamber radius, both the models predict an increase but CRA results in higher values, with ventricular and atrial radii increasing by 10% and 12%, respectively. NCRA predicts a decrease in mean chamber thickness due to the constant $V_{\mathrm{myo}}$ and increased radii, whereas CRA exhibits an increased thickness for all the heart chambers during pregnancy, with the RV thickening by 30% compared with 14% for the LV, whereas the LA and RA thicken by up to 60% and 80%, respectively.

**FIGURE 6:**
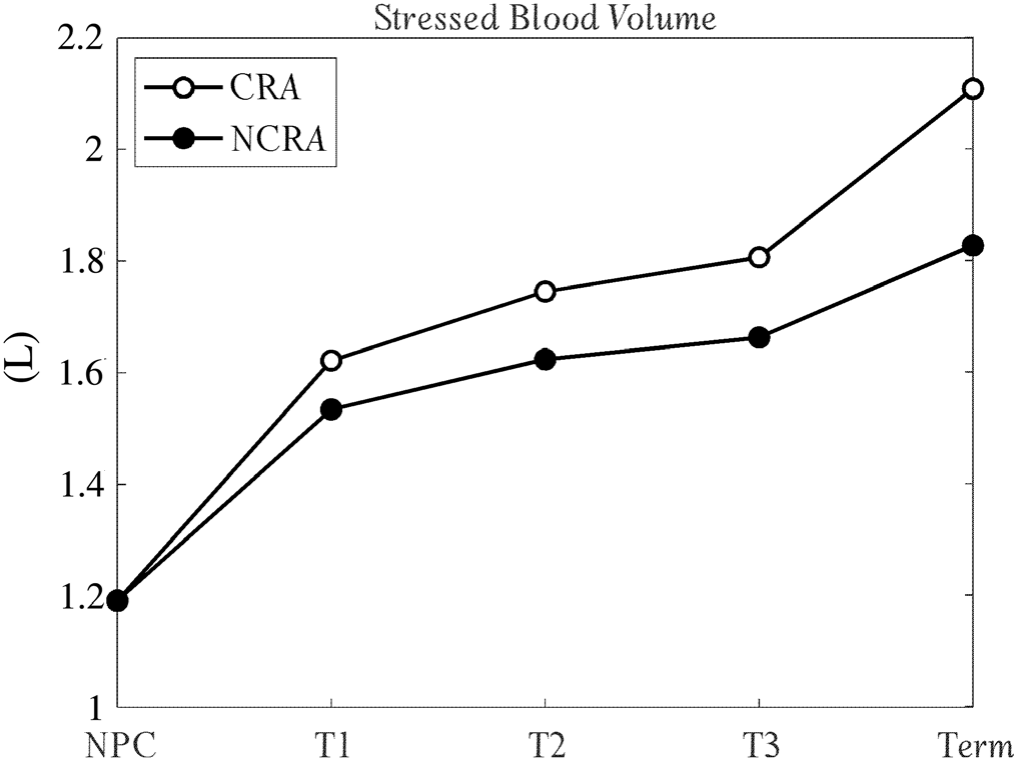
Total stressed blood volume computed for the non‐pregnant case (NPC) and the different trimesters of pregnancy. Black open dotted line, the controlled remodeling algorithm (CRA) simulation and black filled dotted line, the results with no controlled remodeling algorithm (NCRA)

Finally, Figure 6 shows the computed stressed volume for the two models as pregnancy proceeds. Both CRA and NCRA result in increasing $V_{\text{TO}T_{S}}$ from about 1.2 L before pregnancy to about 2.1 and 1.8 L at Term, respectively. Notice that, for the NPC case, by assuming $V_{\text{TO}T_{S}}=30\%V_{\mathrm{TOT}},$ ^50^, ^51^ $V_{\mathrm{TOT}}$ and $V_{\text{TO}T_{U}}$ are about 4 and 2.8 L, respectively. Since the total blood volume at the end of pregnancy is not directly computable, $V_{\text{TO}T_{U}}$ value at Term is assumed to increase of about 0%–33% compared to the NPC value.^52^, ^53^ If $V_{\text{TO}T_{U}}$ does not increase, $V_{\mathrm{TOT}}$ would reach 4.9 L for CRA (representing a 23% increase) and 4.6 L for NCRA (15% increase). On the other hand, if the unstressed volume increases by 33% during pregnancy, $V_{\mathrm{TOT}}$ is predicted to rise by 45% and 38% compared to the NPC value for CRA and NCRA, respectively.

While the above results assumed a constant homeostatic value of $\sigma_{\mathrm{wss}}$ and $\sigma_{f}$ during pregnancy equal to the NPC value, Table S3 reports the outputs of the model when the target values are equal to the upper and lower limits of previously reported values^8^, ^28^, ^46^ (±20%), each value kept constant during pregnancy. Due to the lack of specific clinical data, $\sigma_{\mathrm{wss}}$ is assumed to vary with the same percentage as $\sigma_{f}$. From the table, it is clear that $\sigma_{\mathrm{wss}}$ causes bigger variations compared to $\sigma_{f}$. However, most values are within the reported in vivo ranges. Figure S4 shows the myocardial volume, the radius, and the thickness for the four heart chambers as the pregnancy proceeds and considering the variations of $\sigma_{f}$ and $\sigma_{\mathrm{wss}}$. All the variables vary from the baseline (in green) due to $\sigma_{f}$ or $\sigma_{\mathrm{wss}}$ variation. In particular, an inverse relationship between the fiber/wall shear stress and the volume, radius, and thickness appears: as $\sigma_{f}$ or $\sigma_{\mathrm{wss}}$ increases/decreases $V_{\mathrm{myo}}$, *r*, and *h* decrease/increase.

The results of the global sensitivity analysis are shown in Figure S5 in which we analyzed the behavior of the sensitivity indices for some meaningful outputs: the mean aortic pressure ($mP_{\mathrm{ao}}$), the mean inferior caval venous pressure ($mP_{\mathrm{ivc}}$), the mean pulmonary artery pressure ($mP_{\mathrm{pua}}$), and the CO. Notice that sensitivity indices that resulted lower than 0.05 were put equal to zero, that is, the impact of the corresponding parameters on the uncertainties of the model outputs was considered negligible. Only few inputs have a detectable influence on the selected outputs. In particular, it can be seen that: (i) systemic pressures are mainly influenced by the elastances of the heart with $mP_{\mathrm{ao}}$ affected also by the heart rate and $mP_{\mathrm{ivc}}$ influenced also by the tricuspid valve area; (ii) $mP_{\mathrm{pua}}$ is affected by heart elastances and pulmonary vascular resistances; (iii) CO is influenced by heart elastances and heart rate. In addition, when the inputs vary in the range ±15% the analyzed pressures and flow rate exhibit limited variations which seems acceptable in the cardiovascular panorama.

## 4 DISCUSSION

Pregnancy is a volume overload condition accompanied by substantial hormonal changes and increased demands on the heart.^7^, ^34^ These variations drive a reversible eccentric hypertrophy,^6^, ^7^ whose signaling pathways are not completely understood yet.^16^ The resulting cardiac remodeling is most likely a homeostatic mechanism that maintains mechanical and hemodynamic loads within physiological ranges in which cells optimally function.^67^ Particularly, in this study we explored whether maintaining two simple homeostatic values, that is, myofiber ($\sigma_{f}$) and wall shear ($\sigma_{\mathrm{wss}}$) stresses, could predict cardiac remodeling patterns during a simulated normal pregnancy. Indeed, for the first time, we simulated both hemodynamic and structural changes typical of pregnancy, and to verify our hypothesis of gestational cardiac remodeling induced by homeostasis of $\sigma_{f}$ and $\sigma_{\mathrm{wss}}$, we compared CRA with NCRA. Perhaps surprisingly, even without explicitly representing complex hormonal signaling which may influence cardiac remodeling, using our lumped parameter model we found that these two simple homeostatic principles were able to predict the main cardiac structural changes that appear during pregnancy. This was achieved by starting with our previous work in which a woman‐specific model has been developed,^18^ enforcing reasonable cardiovascular target variables required by the changing physiological demands during pregnancy and applying an updated version of the cardiac remodeling algorithm of Maksuti et al.^17^ From the NPC model, we considered the physiological changes typical of pregnancy by comparing the application of only the hemodynamic changes (NCRA), and the additional impact of fixed cardiac stresses (CRA). However, changes in CO, heart rate, total vascular compliance, and total vascular resistance were prescribed in both the models, therefore implicitly reflecting the effects of hormones, and the altered functions of the sympathetic activity and the RAAS system on the vascular tree.^3^, ^22^, ^23^ On the other hand, homeostatic values of $\sigma_{f}$ and $\sigma_{\mathrm{wss}}$ were imposed only for CRA.

CO and SV are reported to increase by at least 30% and by about 20%–30%,^1^ respectively. Given that CO was a target value and heart rate was prescribed, both NCRA and CRA reflected these basic hemodynamic changes, with achieved SV and CO increases of 22% and 37% for NCRA and 25% and 39% for CRA. Considering the blood pressures, both the models predicted slightly higher values compared to reference data,^28^ however, the values are still within the physiological ranges. The observed differences are likely due to the imposed SVR values, which are slightly higher compared to the reported values.

The imposition of a target CO led to an increase in LAD and LVEDD for both the models, however, only changes seen with CRA agreed with the reported increases at Term of about 15% and 10%, respectively, with differences between computed (CRA) and clinical values for each trimester being less than 5%.^1^, ^28^ Indeed, CRA resulted in LAD and LVEDD increase of about 13% and 9%, respectively; whereas, with NCRA, only 7% and 4% increments were found. Note that differences in absolute values of LAD and LVEDD compared with the reference data^28^ for the NPC output, are likely due to the simplified geometry assumed. Indeed, in our model, the atria and ventricles are simulated as perfect spheres or part of an ellipsoid. This is not true in reality with the heart chambers characterized by a much more complex geometry.

The literature also reports an LV thickness increment of 15%–25% during pregnancy because of the increased preload and afterload, and as an adaptation to minimize the wall stress.^1^, ^8^ This result was seen only with CRA (increase of about 14%) whereas NCRA resulted in an LV thickness reduction (of about 3%), which is not representative of normal physiological remodeling with pregnancy.^6^, ^7^, ^68^, ^69^ Moreover, as expected, only CRA determined an LVM increase of about 40% that agrees well with the in vivo augmentation of about 50%.^1^, ^3^, ^28^, ^29^ In addition, LVM in T1 (CRA) increased by only 4% compared to the NPC value, and this agrees with the lack of alteration found in Reference 24. Also the mass of the RV is found to increase by about 40% in the third trimester,^29^ in agreement with our CRA findings at T3. Concerning RWT, the models predicted an almost constant value, in agreement with the features of eccentric hypertrophy.^7^, ^67^ The reference values, on the other hand, exhibit a bigger increase toward the end of pregnancy, with Melchiorre et al.^28^ reporting emergence of cardiac maladaptation (i.e., increased wall stress) near term in apparently healthy women, a finding not reproduced by our model due to the imposed assumption of a constant wall stress. This is further confirmed by the remodeling categorization shown in Figure 6 of Lang et al.^70^ based on RWT, which shows that, in all the trimesters for both the models, the ventricle would be considered to have a “normal geometry” relative to the increasing BSA,^70^ in agreement with the simulation of physiological healthy pregnancy.

To further validate the model, we also computed the total vascular blood volume ($V_{\mathrm{TOT}}$) as the sum of the stressed ($V_{\text{TO}T_{S}}$) and the unstressed ($V_{\text{TO}T_{U}}$) vascular volume. $V_{\text{TO}T_{S}}$ is directly computed from simulations as the sum of the volume of the different compartments of Figure 1, whereas $V_{\text{TO}T_{U}}$ required more attention, at least during pregnancy. Indeed, for NPC, it is known that $V_{\text{TO}T_{U}}≅25\%–30\%$ of $V_{\mathrm{TOT}}.$ ^50^, ^51^ This distribution resulted in $V_{\mathrm{TOT}}=$ 4 L that is within the normal ranges given by Wadsworth.^71^ As pregnancy proceeds, the increase in the unstressed volume is well recognized,^54^, ^55^, ^56^ however, how much this increases is not clear. Experimental data is conflicting, but suggests that unstressed volume remains constant or increase by up to 33%.^52^, ^53^ By assuming these as lower and upper limits, the total blood volume in our simulations increased by 23%–45% for CRA and 15%–38% for NCRA. These ranges reasonably agree with the 20%–100% increase of blood volume (“usually close to 45%”) reported by Sanghavi and Rutherford^3^ and the 50% increase stated by Edouard et al.,^57^ further confirming the validity of the CRA model.

Considering now the effects of stress values, remodeling based on constant homeostatic values of $\sigma_{f}$ and $\sigma_{\mathrm{wss}}$ seems to closely replicate the cardiac changes that occur during pregnancy. Variations of the prescribed values within previously reported values (±20%)^8^, ^28^, ^46^ do alter the absolute values of the heart structure, but within physiological ranges. Particularly, a bigger $\sigma_{f}$ (or $\sigma_{\mathrm{wss}}$) value led to smaller $V_{\mathrm{myo}}$, *r*, and *h*, and vice versa, consistent with expectations. Indeed, this behavior is expected for two reasons: (i) there is a direct proportional relation between myofiber stress and pressure, and wall shear stress and flow; and (ii) cardiac remodeling is a complex process, which increases $V_{\mathrm{myo}}$, *r*, and *h* when cardiac stresses are higher than the physiological “set‐point,” that is, cardiac remodeling restores homeostatic value of stresses. Thus, for example, when $\sigma_{f}$=53 mmHg is considered as (homeostatic) physiological target, hemodynamic conditions causing $\sigma_{f}$=64 mmHg triggers the remodeling by increasing $V_{\mathrm{myo}}$, *r*, and *h* and restoring $\sigma_{f}$ to the physiological value. On the other hand, if $\sigma_{f}$= 64 mmHg is the homeostatic target, cardiac remodeling is not triggered and physiological (hence lower) values of $V_{\mathrm{myo}}$, *r*, and *h* are maintained. The opposite happens if lower stress values are considered.

To the best of our knowledge, this is the first attempt to simulate both the hemodynamic and geometrical changes that occur in the maternal circulation. We demonstrated that although NCRA is able to replicate pregnant hemodynamics, it cannot capture cardiac remodeling. On the other hand, CRA was able to predict the altered gestational hemodynamics and the physiological remodeling typical of pregnancy, giving a first verification of our hypothesis on regarding the homeostatic role of the stresses. For this reason, CRA may be useful for studying remodeling during both healthy and abnormal pregnancies.

### 4.1 Clinical implications and future development

The CRA model was able to reproduce key physiological changes that occur during pregnancy and may be a useful tool to better understand the adaptive process that occur in the cardiovascular system during pregnancy. Indeed, the results suggest that those signaling pathways involved with $\sigma_{f}$ and $\sigma_{\mathrm{wss}}$ may be of great importance in gestational cardiac remodeling. This may be useful for understanding hemodynamics and associated cardiac function and remodeling in high‐risk pregnancies (such as gestational hypertension or congenital heart disease), although application to such questions would require additional model validation. Moreover, further work is needed to incorporate the hormonal modulation of remodeling and to assess its impact in different settings.

### 4.2 Limitations

The developed model is based on a simplified heart geometry, which may limit future applications in settings such as complex congenital heart disease. The NPC absolute values of LAD and LVEDD were higher than the reference values, although we showed that the percentage changes during pregnancy agreed very closely with the literature. In addition, we assumed that $\sigma_{\mathrm{wss}}$ and $\sigma_{f}$ constitute fixed homeostatic “target” values for the remodeling process. Although these two simple assumptions resulted in an excellent prediction of changes in cardiac geometry, compared with literature data, in reality it is likely that cardiac remodeling is affected by other factors. For example, genetic factors are known to play a key role in some forms of pathological remodeling, as well as hormones.^72^, ^73^ Moreover, the application of Equation (3) to half and quarter of ellipsoidal ventricles is a limitation of the present work. However, we use this simple model as a first‐order approximation and since it performs well it may be speculated that the assumptions do not introduce major issues. Moreover, the used model is equivalent to the one applied in clinical practice; thus, it allows the immediate comparison between computed and clinical data. Note also that, $\sigma_{\mathrm{wss}}$ was computed assuming a laminar flow of Poiseuille's type through a cylindrical pipe. This choice was used as a pragmatic albeit gross first approximation, but this does not strictly apply to the geometries considered or if flow conditions are turbulent.

## 5 CONCLUSIONS

We developed a lumped parameter model of the whole circulation able to reproduce the hemodynamic changes that arise during pregnancy, and incorporated a cardiac remodeling algorithm based wall stress and chamber wall shear stress homeostasis principles. Although employing two very simple assumptions, this algorithm predicted changes in cardiac mass and geometry that were very similar to those reported in normal human pregnancies. These techniques provide insight into the biomechanical basis of cardiac remodeling during pregnancy and may be useful in future to investigate cardiovascular problems that arise in some pregnancies.

## CONFLICT OF INTEREST

All the authors declare that they have no conflict of interest. Jonathan P. Mynard is a consultant to the Brain Protection Company, Tournicare, Masimo Corporation and Baxter Healthcare; none of these consultancies have any relation to the present work.

## AUTHOR'S CONTRIBUTIONS

All authors contributed to the study conception and design. Material preparation, data collection, and analysis were performed by Giulia Comunale, Francesca M. Susin, and Jonathan P. Mynard. The first draft of the manuscript was written by Giulia Comunale and all authors commented on previous versions of the manuscript. All authors read and approved the final manuscript.

## Supporting information


**Data S1.** Supporting information.

## Data Availability

The data produced and analyzed during the current study are available from the corresponding author on reasonable request.
