# Supplementary material for: Ventricular wall stress and wall shear stress homeostasis predicts cardiac remodeling during pregnancy: A modeling study
Source: Int J Numer Method Biomed Eng. 2021 Oct 18;38(1):e3536. doi: 10.1002/cnm.3536 (PMC9285413; doi:10.1002/cnm.3536)
Supplement: Supplementary file 1 — Data S1. Supporting information. [file CNM-38-e3536-s001.docx]

**Ventricular wall stress and wall shear stress homeostasis predicts cardiac remodeling during pregnancy: A modelling study**

Giulia Comunale^1, 2^, Francesca M. Susin^1^, Jonathan P. Mynard^2,3,4^.

^1^ Cardiovascular Fluid Dynamics Laboratory HER, Department of Civil, Environmental and Architectural Engineering – University of Padova, Italy.

^2^ Heart Research, Murdoch Children’s Research Institute, Parkville VIC, Australia.

^3^ Department of Pediatrics, University of Melbourne, Parkville VIC, Australia.

^4^ Department of Biomedical Engineering, University of Melbourne, Parkville VIC, Australia.

### Supplementary Information

# Methods

*Hemodynamic model*

To simulate the blood circulation, we used the lumped parameters methodology, which enables one to represent pressure and flow rate in the vascular compartments of the body, i.e., any vascular segment, region or organ. Pressures and flows are obtained by computing the Navier-Stokes and mass conservation equations averaged over the three dimensions [74]. The vascular compartments are described by lumped parameters able to replicate the geometrical and physical features. Resistances (*R*) replicate the resistance to flow due to blood viscosity, compliances (*C*) reproduce the elastic properties of the vessels and, the inductances (*L*) reflect the flow inertia. By the combination of these, any section of the vascular system can be considered. Particularly, supplementary Fig. S1 represents an example of compartment, described by

| $\left\{ \begin{aligned} C\frac{dP_{j}}{dt}=Q_{j}-Q_{j+1} \\ L\frac{dQ_{j+1}}{dt}=P_{j}-P_{j+1}-RQ_{j+1} \end{aligned} \right.$ | (S1) |
| --- | --- |

where ($P_{j}$, $Q_{j}$) and ($P_{j+1}$,$Q_{j+1}$) are the upstream and downstream pressures and flow rates, respectively. Particularly, in the present work, the great vessels were represented by supplementary Fig. S1 whereas for the organs of interest we considered the compartment shown in supplementary Fig. S2.

*Geometrical model*

The geometry of each chamber is reported in supplementary Fig. S3. The atrial volumes were computed as

| $\left\{ \begin{aligned} V_{lumen_{a}}=\frac{4\pi r^{3}}{3} \\ V_{myo_{a}}=\frac{4\pi{(r+h)}^{3}}{3}-\frac{4\pi r^{3}}{3} \end{aligned} \right.$ | (S2) |
| --- | --- |

where $V_{lumen_{a}}$ represents the atrial blood (fluid) volume whereas $V_{myo_{a}}$is the atrial myocardial volume, i.e., of the wall. In the same way, we computed the left ($V_{lv}$, $V_{myo_{lv}}$) and right ($V_{rv}$, $V_{myo_{rv}}$) fluid ($V_{lumen}=V_{lv} or V_{rv}$) and wall ventricular volumes ($V_{myo_{lv}}$ and $V_{myo_{rv}}$) considering Eq. (S3) and (S4), respectively.

| $\left\{ \begin{aligned} V_{lv}=2\pi r^{3} \\ V_{myo_{lv}}=\frac{1}{2}\frac{4\pi(3r+h){(r+h)}^{2}}{3}-2\pi r^{3} \end{aligned} \right.$ | (S3) |
| --- | --- |
| $\left\{ \begin{aligned} V_{rv}=\pi r^{3} \\ V_{myo_{rv}}=\frac{\pi(3r+h){(r+h)}^{2}}{3}-\pi r^{3} \end{aligned} \right.$ | (S4) |

Note that Eq. (S2) – (S4) are computed after the hemodynamic model reaches the periodic steady state. In particular, given the heart chamber volume and the myocardial volume, the radius and the thickness of each cardiac chamber can be calculated by proper rearrangement of Eq. (S2) – (S4). Moreover, since $V_{lumen}$ varies over the heartbeat, $r$ and $h$ are also functions of time from which the desired quantities can be extracted, e.g., end-systolic values, mean values, etc.

*Clinical variables*

Here we report the clinical variables considered and the way they were computed. The systolic (SBP), diastolic (DBP), and mean (MAP) blood pressures are the maximum, minimum, and mean values of the aortic pressure, respectively. Stroke volume (SV) was computed as the integral of mitral valve flow over one heartbeat and the cardiac output as $CO=SV\cdot HR$. The total systemic vascular resistance (SVR) was computed as $SVR={(MAP}/{CO)\cdot80}$, the cardiac work (CW) is $CW=CO\cdot MAP$, and the relative wall thickness (RWT) is the ratio between the chamber’s thickness and diameter at end diastole, *h_ED_* and LVEDD, respectively [8,28]. Finally, the left atrial diameter (LAD) is the mean diameter computed over one heartbeat.

# Figures

**Fig. S1** Example of lumped parameter compartment. *R*, resistance, *C*, compliance, *L*, inductance, ($P_{j}$, $Q_{j}$) and ($P_{j+1}, Q_{j+1}$) the upstream and downstream pressures and flow rates, respectively.

*

*

**Fig. S2** Lumped parameter compartment used in the present work to replicate the organs of interest. The arterial and venous sides are composed by resistance and compliance ($R_{art}, C_{art}$) and ($R_{art}, C_{art}$), respectively, whereas the vascular bed was replicated by a resistance ($R_{vb}$).





**Fig. S3** Geometrical model of *a)* atria, *b)* left ventricle, and *c)* right ventricle. Each chamber has a constant thickness of *h*, and an inner radius of *r*. The ventricular longitudinal dimension is *3r*.


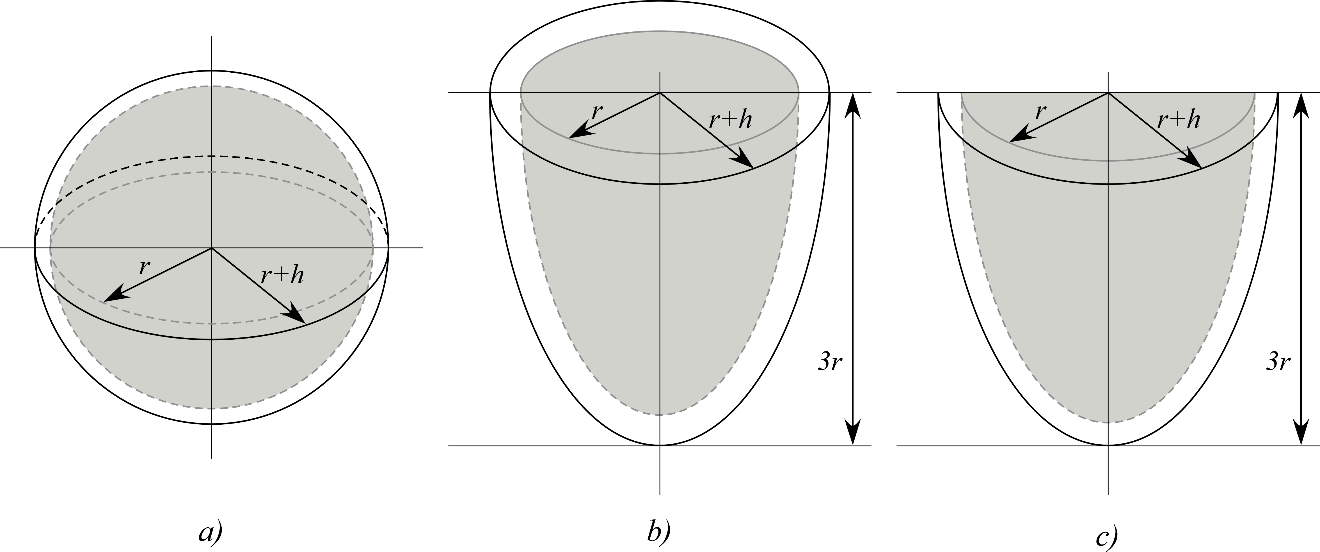


**Fig. S4** Outputs of the controlled remodeling algorithm (CRA) model by changing separately $\sigma_{f}$ and $\sigma_{wss}$. $\sigma_{f}$ changes within physiological ranges, whereas, due to the lack of clinical data, $\sigma_{wss}$ was changed by applying the same percentage change as $\sigma_{f}$. The results are reported for the four heart chambers for the different trimesters of pregnancy. In the first row, the myocardial volume, in the second row, the mean chamber’s radius, and in the third row, the mean chamber’s thickness. In green, the CRA simulation, in magenta, ${\sigma_{f}}_{UT}$ (upper threshold), in red, ${\sigma_{f}}_{LT}$ (lower threshold), in blue, ${\sigma_{wss}}_{UT}$ (upper threshold), and in light blue, ${\sigma_{wss}}_{LT}$ (lower threshold).


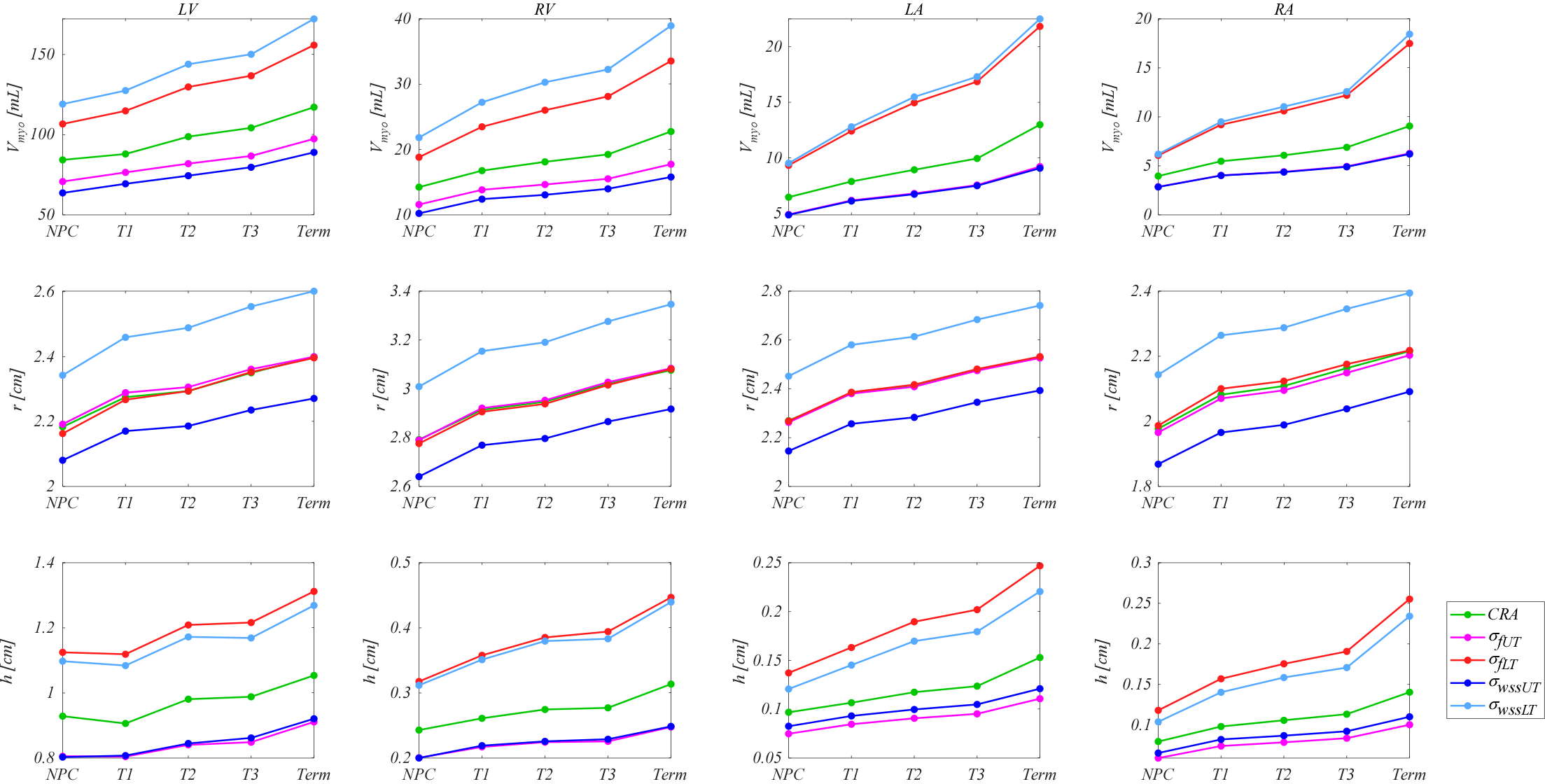


**Fig. S5** Computed global sensitivity indices. $mP_{ao}$, the mean aortic pressure, $mP_{ivc}$, the mean inferior caval venous pressure, $mP_{pua}$, the mean pulmonary artery pressure, and $CO$, the cardiac output. $A_{aov}$, aortic valve area, $A_{mv}$, mitral valve area, $A_{pv}$, pulmonary valve area, $A_{tv}$, tricuspid valve area, $E_{{max}_{LA}}$, LA maximum elastance, $E_{{max}_{LV}}$, LV maximum elastance, $E_{{max}_{RA}}$, RA maximum elastance, $E_{{max}_{RV}}$, RV maximum elastance, $E_{{min}_{LA}}$, LA minimum elastance, $E_{{min}_{LV}}$, LV minimum elastance, $E_{{min}_{RA}}$, RA minimum elastance, $E_{{min}_{RV}}$, RV minimum elastance, HR, heart rate, $L_{ao}$, aortic inductance, $L_{ivcab}$, abdominal inferior vena cava inductance, $L_{ivcth}$, thoracic inferior vena cava inductance, $L_{pua}$, pulmonary arterial inductance, $L_{puve}$, pulmonary venous inductance, $L_{svc}$, superior vena cava inductance, $m_{1_{LA}}$, LA systolic steepness, $m_{1_{LV}}$, LV systolic steepness, $m_{1_{RA}}$, RA systolic steepness, $m_{1_{RV}}$, RV systolic steepness, $m_{2_{LA}}$, LA diastolic steepness, $m_{2_{LV}}$, LV diastolic steepness, $m_{2_{RA}}$, RA diastolic steepness, $m_{2_{RV}}$, RV diastolic steepness, SVR, systemic vascular resistance, PVR, pulmonary vascular resistance, $C_{{sys}_{A}}$, total systemic arterial compliance, $C_{{sys}_{V}}$, total systemic venous compliance, $C_{{pul}_{A}}$, total pulmonary arterial compliance, $C_{{pul}_{V}}$, total pulmonary venous compliance.


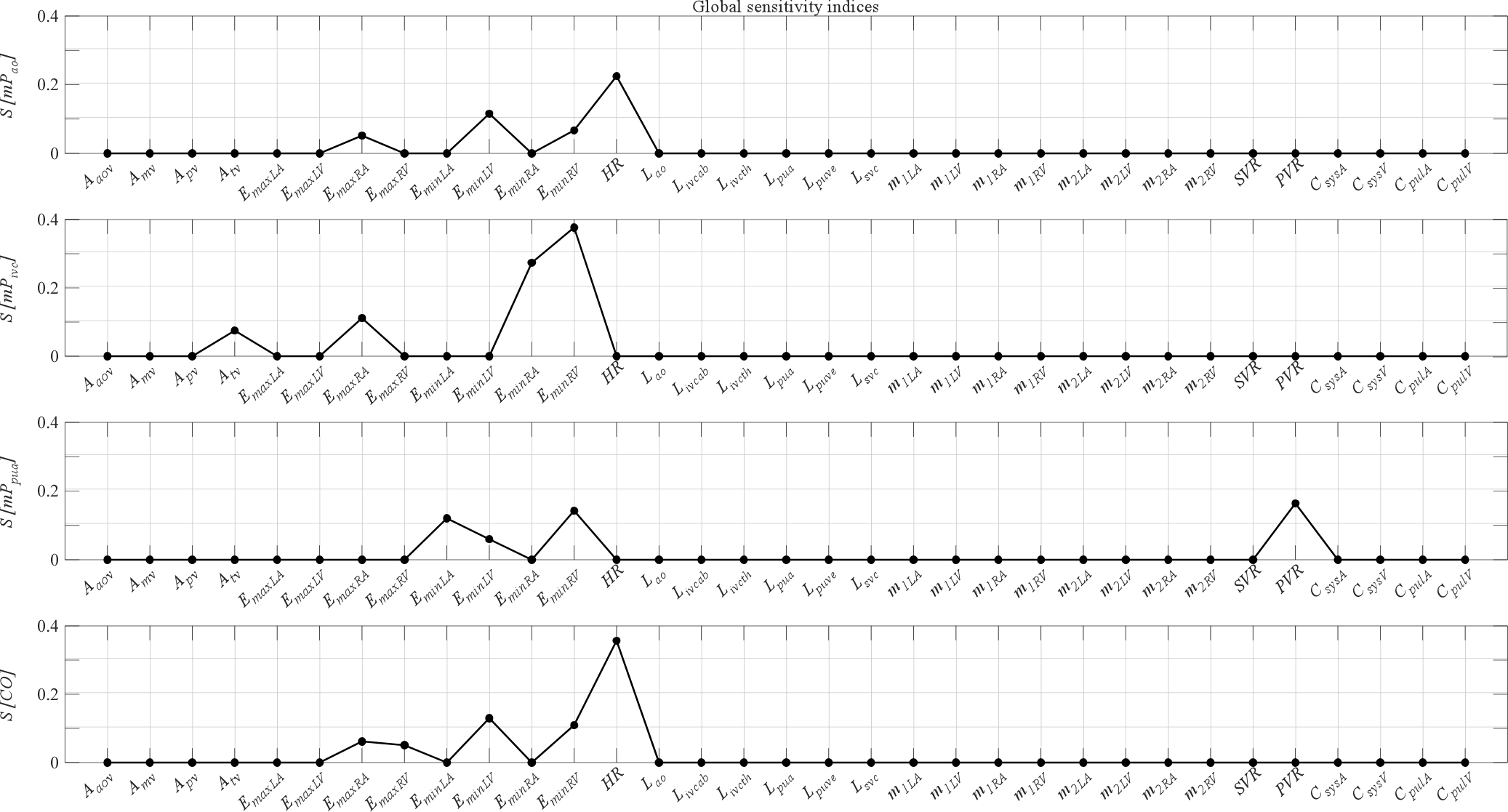


# **Tables**

Table S1 Percentage changes in total vascular resistance and total systemic vascular compliance during gestation. Variations are given with respect to NPC and in according to [1].

| **Parameter** | **T1** | **T2** | **T3** | **Term** |
| --- | --- | --- | --- | --- |
| SVR | -5% | -4% | -15% | -8% |
| Ctsv | 30% | 30% | 30% | 30% |

NPC, non-pregnant case, T1, first trimester, T2, second trimester, T3, third trimester and Term, end of pregnancy. SVR, total systemic vascular resistance and Ctsv, total systemic vascular compliance.

Table S2 Myofiber and wall shear stresses computed by the models for each heart chamber in the non-pregnant case (NPC) configuration.

| **Parameters** | **LV** | **RV** | **LA** | **RA** |
| --- | --- | --- | --- | --- |
| $\sigma_{f} [mmHg]$ | 52 | 58 | 81 | 42 |
| $\sigma_{wss} [mmHg]$ | 0.0026 | 0.0012 | 0.0023 | 0.0036 |

**Table S3** Percentage variations of the controlled remodeling algorithm (CRA) model by changing separately the myofiber stress ($\sigma_{f}$) and wall shear stress ($\sigma_{wss}$). $\sigma_{f}$ changes within physiological ranges, whereas, due to the lack of clinical data, $\sigma_{wss}$ was changed by applying the same percentage change as $\sigma_{f}$. The outputs are reported as percentage variation and ranges due $\sigma_{f}$ or $\sigma_{wss}$ change in parenthesis (if the parameter changes).

| **Parameters** | **T1** | | **T2** | | **T3** | | **Term** | |
| --- | --- | --- | --- | --- | --- | --- | --- | --- |
|  | $\sigma_{f}$ | $\sigma_{wss}$ | $\sigma_{f}$ | $\sigma_{wss}$ | $\sigma_{f}$ | $\sigma_{wss}$ | $\sigma_{f}$ | $\sigma_{wss}$ |
| SVR [dynes/s per cm^5^] | 2% (1089-1110) | 3% (1086-1118) | 2% (1121-1148) | 4% (1117-1157) | 3% (992-1020) | 4% (989-1028) | 4% (1024-1066) | 6% (1020-1078) |
| SBP [mmHg] | 3% (102-105) | 5% (101-106) | 4% (108-113) | 6% (108-114) | 4% (105-110) | 6% (104-111) | 6% (116-122) | 8% (115-124) |
| DBP [mmHg] | 1% (65-66) | 1% (65-66) | 1% (70-71) | 2% (70-71) | 1% (67-68) | 2% (66-68) | 3% (73-75) | 4% (73-75) |
| MAP [mmHg] | 2% (78-79) | 3% (77-80) | 2% (83-85) | 4% (82-85) | 3% (79-82) | 4% (79-82) | 4% (87-91) | 6% (87-92) |
| CO [L/min] | 0% | 0% | 0% | 0% | 0% | 0% | 0% | 0% |
| EDV [mL] | 0% | 26% (107-138) | 0% | 26% (110-142) | 0% | 27% (115-150) | 0% | 27% (124-161) |
| ESV [mL] | 0% | 75% (30-60) | 0% | 75% (31-63) | 0% | 72% (36-70) | 0% | 73% (37-74) |
| SV [mL] | 0% | 0% | 0% | 0% | 0% | 0% | 0% | 0% |
| EF [%] | 0% | 24% (55-71) | 0% | 25% (55-71) | 0% | 26% (52-68) | 0% | 25% (53-69) |
| CW [mmHg L per min] | 2% (442-451) | 3% (441-454) | 2% (488-499) | 4% (486-503) | 3% (508-522) | 4% (506-526) | 4% (592-616) | 6% (589-623) |
| LAD [cm] | 0% | 14% (4.5-5.2) | 0% | 14% (4.6-5.2) | 0% | 14% (4.7-5.4) | 0% | 14% (4.8-5.5) |
| LVEDD [cm] | 1% (5.3-5.3) | 10% (5.1-5.6) | 1% (5.3-5.3) | 10% (5.1-5.6) | 1% (5.4-5.4) | 10% (5.2-5.7) | 0% | 9% (5.4-5.9) |
| LVM [g] | 44% (80-119) | 66% (72-133) | 48% (85-135) | 70% (77-150) | 48% (90-142) | 68% (83-156) | 50% (101-162) | 71% (93-179) |
| RWT [-] | 37% (0.24-0.34) | 30% (0.24-0.32) | 38% (0.25-0.36) | 34% (0.25-0.35) | 38% (0.25-0.36) | 28% (0.26-0.34) | 40% (0.26-0.38) | 33% (0.26-0.36) |

See Table 3 for abbreviation

# Additional references

74. Formaggia L, Veneziani A. Reduced and multiscale models for the human cardiovascular system. From notes on *7^th^ VKI Lecture Series on “Biological Fluid Dynamics”* held at the Von Karman Institute, Belgium, 2003; (May). doi:10.13140/RG.2.1.3668.8088.
